# Association between chronic diseases and depression in the middle-aged and older adult Chinese population—a seven-year follow-up study based on CHARLS

**DOI:** 10.3389/fpubh.2023.1176669

**Published:** 2023-07-20

**Authors:** Pengfei Zhou, Shuai Wang, Ya Yan, Qiang Lu, Jiaxing Pei, Wang Guo, Xiaoguang Yang, Yunming Li

**Affiliations:** ^1^Department of Information, Medical Support Center, The General Hospital of Western Theater Command, Chengdu, Sichuan, China; ^2^School of Public Health, Southwest Medical University, Luzhou, Sichuan, China; ^3^Department of Outpatient, The General Hospital of Western Theater Command, Chengdu, Sichuan, China; ^4^Department of Statistics, College of Mathematics, Southwest Jiaotong University, Chengdu, Sichuan, China

**Keywords:** depression, aging, chronic disease, Chinese, mental health

## Abstract

**Background:**

With the aging of the Chinese population, the prevalence of depression and chronic diseases is continually growing among middle-aged and older adult people. This study aimed to investigate the association between chronic diseases and depression in this population.

**Methods:**

Data from the China Health and Retirement Longitudinal Study (CHARLS) 2011–2018 longitudinal survey, a 7-years follow-up of 7,163 participants over 45 years old, with no depression at baseline (2011). The chronic disease status in our study was based on the self-report of the participants, and depression was defined by the 10-item Center for Epidemiologic Studies Depression Scale (CES-D-10). The relationship between baseline chronic disease and depression was assessed by the Kaplan–Meier method and Cox proportional hazards regression models.

**Results:**

After 7-years follow-up, 41.2% (2,951/7163, 95% *CI*:40.1, 42.3%) of the participants reported depression. The analysis showed that participants with chronic diseases at baseline had a higher risk of depression and that such risk increased significantly with the number of chronic diseases suffered (1 chronic disease: *HR* = 1.197; 2 chronic diseases: *HR* = 1.310; 3 and more chronic diseases: *HR* = 1.397). Diabetes or high blood sugar (*HR* = 1.185), kidney disease (*HR* = 1.252), stomach or other digestive diseases (*HR* = 1.128), and arthritis or rheumatism (*HR* = 1.221) all significantly increased the risk of depression in middle-aged and older adult Chinese.

**Conclusion:**

The present study found that suffering from different degrees of chronic diseases increased the risk of depression in middle-aged and older adult people, and these findings may benefit preventing depression and improving the quality of mental health in this group.

## 1. Introduction

Aging is one of the most serious public health problems in China, and the old-age population in China is greater than the combined older adult populations of all the European countries (1). As of 2020, China has more than 264 million people over 60 years old, accounting for 18.7% of the country’s population, and the average life expectancy has increased from 55.8 years in 1953 to 77.9 years in 2020 (2). Age-related health problems are one of the most severe consequences of population aging, such as chronic diseases in middle-aged and older adult people. Chronic diseases are socially harmful and may impose an enormous psychological and economic burden on patients and their families (3). It has been reported that chronic diseases have become the most significant health problem for the Chinese population and are the most prominent factor leading to the years lived with disability (YLD) (4). As the aging process accelerates, the prevalence of chronic diseases among the older adult population continues to increase (5). An epidemiological study of Chinese older adults found that 75.8% suffered from at least one chronic disease (6), and the risk of suffering two or more chronic diseases at the same time increases significantly with aging (7, 8).

Depression is a serious mental illness that causes abnormal moods, insomnia, loss of interest in life, and suicidal tendencies. It is estimated that more than one million people worldwide commit suicide yearly due to depression (9, 10). In recent years, the number of people with depression has been climbing. In 2017, the number of people with depression worldwide reached 258 million, of which in China exceeded 56 million (21.3%) (10, 11). The World Health Organization (WHO) predicts that depression will be the most burdensome disease in the world by 2030 (12). The middle-aged and older adult population is a high-risk population for depression, and the prevalence of depression increases with age. The accumulation of factors such as illness and loss of family and friends may exacerbate their emotional distress, leading to a greater susceptibility to negative emotions such as anxiety and depression, and some previous studies have found that the prevalence of depression in the middle-aged and older adult population in China is about 17.4 to 46.15% (13–16).

Some studies have shown that chronic diseases are strongly associated with depression (14, 15), including diabetes (17–19), chronic liver disease (20), kidney disease (21), cancer (22), stroke (23), and chronic obstructive pulmonary disease (COPD) (24), and as the number of chronic diseases increases, the patients have more severe mental disorders and a significantly increased the risk of depression (9, 25). In addition, several sociodemographic factors, as well as lifestyle factors such as age, education, BMI, marital status, economic status, social activities, nighttime sleep duration, and smoking or alcohol consumption, have also been shown to have a possible association with the development of depression (9, 26, 27), and there may exist some interaction effects between these factors. Therefore, exploring the risk factors for depression in middle-aged and older adult people and their interaction effects, as well as the relationship between depression and chronic diseases is significant for preventing and treating depression. Several previous studies used the CHARLS database to investigate the association between chronic diseases and depression in the Chinese middle-aged and older adult population, and they discovered a significant association between chronic diseases and depression, as well as that populations with multiple chronic diseases were at higher risk for depression (9, 15, 27–30).

However, because the majority of these studies are cross-sectional, causal conclusions about the association between chronic illnesses and depression cannot be drawn (15, 28–30). Previous cohort studies with short follow-up did not evaluate the most recent available data from CHARLS, and the study conclusions were out of date (9, 27). Furthermore, depression develops slowly, and insufficient follow-up time may result in an inaccurate judgment. Therefore, long-term cohort studies with the latest data are necessary to validate the possible causal association between common chronic diseases and depression.

Therefore, the current study aimed to analyze the latest causal relationship between depression and common chronic diseases in the population aged 45 years and older using a cohort data (2011–2018) from the China Health and Retirement Longitudinal Study (CHARLS) (31). Specifically, this study focused on the following issues: 1) whether having chronic diseases increases the risk of depression in middle-aged and older adults compared to those who do not have chronic diseases, 2) the relationship between the risk of depression and the number of chronic diseases suffered, and whether there are differences in the influencing factors of depression and its risk after stratification according to regions, 3) which specific chronic diseases increase the risk of depression, and 4) whether there are interaction effects of social activity participation, age, and chronic disease status on the incidence of depression.

## 2. Materials and methods

### 2.1. Population

The data for this study were obtained from the CHARLS, an extensive interdisciplinary survey funded by Peking University, which aims to collect a set of high-quality microdata broadly representative of individuals and households of the middle-aged and older adult population over 45 years old in China (32). The information includes basic personal information, health status, income and asset status, etc. It is used to analyze the aging situation of the Chinese population, promote interdisciplinary research on the aging issues, and provide a more scientific foundation for formulating and improving relevant policies in China. CHARLS used stratified sampling and Probability-Proportional-to-Size sampling (PPS) to conduct follow-up surveys in 150 counties and 450 communities (villages) in 28 provinces (autonomous regions and municipalities directly under the central government) in 2011, 2013, 2015, and 2018, respectively. As such, CHARLS data are widely representative and reflect the overall situation of the middle-aged and older adult groups in Chinese urban and rural areas.

**Figure 1:**
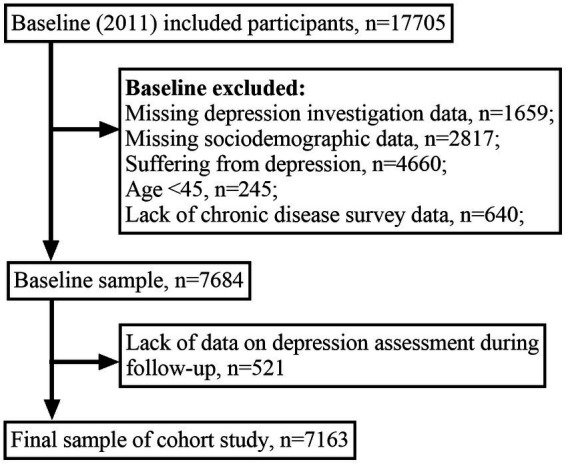
Process of sample population screening.

This study used data from four survey studies, 2011, 2013, 2015, and 2018, with a baseline data (2011) of 17,705 individuals. According to the design of the present study, 2,817 individuals were excluded due to missing essential information (age, gender, education, marital status, etc.) in the baseline data, 1,659 were excluded because of the lack of a depression survey at baseline, 640 were excluded because of missing information in the chronic disease survey, and 245 were eliminated because they were below 45 years of age. In addition, this study used a cohort study design that required the removal of respondents who were already suffering from depression at baseline (2011), so 4,660 participants were excluded from our study, and 521 were excluded due to a lack of data on depression assessment during follow-up. Therefore, 7,163 participants were finally included in this study for analysis. Figure 1 shows the screening process of the sample population.

### 2.2. Assessment of the depression

The depression of the respondents in our study was assessed using the 10-item Center for Epidemiologic Studies Depression Scale (CES-D-10) (33, 34). Previous research has shown that the CES-D-10 demonstrated excellent internal consistency (Cronbach *α* = 0.69–0.89), sensitivity (71.4–84.6%), and specificity (72.6–95%) for depression screening (35). In addition, the CES-D-10 has been fully validated in the Chinese older adult population to demonstrate its satisfactory reliability and validity (36, 37). The scale evaluates the respondent’s psychological situation in the past week. It comprises questions in 10 aspects: “I was bothered by things that do not usually bother me,” “I had trouble keeping my mind on what I was doing,” “I felt depressed,” “I felt everything I did was an effort,” “I felt hopeful about the future,” “I felt fearful,” “My sleep was restless,” “I was happy,” “I felt lonely,” and “I could not get going.” The choices were “Rarely or none of the time (<1 day),” “Some or a little of the time (1–2 days),” “Occasionally or a moderate amount of the time (3–4 days),” and “most of the time (5–7 days),” which were scored 0 ~ 3, and two questions reflecting positive sentiment (I felt hopeful about the future, I was happy) were achieved in reverse (3 ~ 0). The final score was calculated by adding the scores of the ten questions. The total scores ranged from 0 to 30, and the participants were considered with depression when the total score was ≥10 (9, 38, 39).

### 2.3. Assessment of the chronic diseases

The chronic diseases were determined by a surveyor using the question “Have you been diagnosed with … by a doctor,” and the participants were asked each of the following 13 categories of chronic diseases: hypertension, dyslipidemia (elevation of low-density lipoprotein, triglycerides (TGs), and total cholesterol, or a low high-density lipoprotein level), diabetes or high blood sugar, cancer or malignant tumor (excluding minor skin cancers), chronic lung diseases [such as chronic bronchitis, emphysema (excluding tumors or cancer)], liver disease (except fatty liver, tumors, and cancer), heart attack, coronary heart disease, angina, congestive heart failure, or other heart problems, stroke, kidney disease (except for tumor or cancer), stomach or other digestive diseases (except for tumor or cancer), memory-related disease, arthritis or rheumatism, asthma. For each of these chronic diseases, respondents were given a score of “1” if they reported having an illness and “0” if they did not. Finally, the chronic disease scores were added up to the number of chronic diseases suffered by the respondents, and the number of chronic diseases ≥2 was considered “multimorbidity” (8).

### 2.4. Other covariates

The covariates included age, gender, the highest level of education, marital status, health insurance status, nighttime sleep duration, drinking status, smoking status, social activity participation, income status, body mass index (BMI), and region. Existing research demonstrates that these variables might be associated with depression (14, 40). In our study, data for chronic diseases and covariates were obtained from the baseline investigation (2011), and depression assessment data were collected from each follow-up survey (2011–2018). Appendix 1 shows the code for all variables and their problem descriptions.

In our study, we categorized participants’ highest level of education into primary school and below, middle school, high school and above. Marital status was separated into married, divorced, widowed, and unmarried. The types of health insurance in the original survey data were classified as urban and rural resident medical insurance, long-term care insurance, urban employee medical insurance, private medical insurance and government medical insurance, etc. In this study, a respondent was considered covered by medical insurance if they participated in any of these. Participants were interviewed about their sleep status using the question “During the past month, how many hours of actual sleep did you get at night (average hours for one night),” and we grouped the hours of sleep reported by participants into <5 h, 5–8 h and > 8 h. Drinking status was divided into greater than once a month, less than once a month and never. Smoking status was categorized as current smoker, former smoker and never smoked. The question “Have you done any of these activities in the last month” was used to ask participants about social activities, including interacting with friends, doing voluntary or charity work, using the internet, and other types of social activities, etc. Respondents who engaged in any of these social activities were considered socially active. BMI was divided into four groups: less than 18.5 kg/m^2^ (underweight), 18.5 ~ 24 kg/m^2^ (normal weight), 24 ~ 28 kg/m^2^ (overweight), and more than 28 kg/m^2^ (obese) (41, 42). The region was divided into Eastern, Central, and Western, and because of the small sample size of three provinces in the Northeast (Jilin, Heilongjiang, and Liaoning), we included them in the Central region for analysis.

### 2.5. Statistical analysis

The Mean ± standard deviation (*x̅* ± SD) was used for the statistical description of the continuous numerical variables, and frequency (*n*) and percentage (%) were used for the statistical description of the categorical variables in our study. The Kaplan–Meier (K-M) method and Cox proportional hazards regression models were used to explore the association between each baseline characteristic, chronic diseases, and depression. The trend tests were used to analyze the change in the risk of depression with the increasing number of chronic diseases.

Firstly, the univariate Cox proportional hazards regression model was used to assess the association of each baseline covariate with depression and to estimate the hazard ratio (*HR*) and its 95% confidence interval (95% *CI*). The Kaplan–Meier method was then adopted to evaluate relationship between the chronic diseases and depression and to plot the incidence curves. After including each covariate, the association between the number of chronic diseases and depression was explored using a multivariate Cox proportional hazards regression model, adjust *HR* and 95% *CI* were calculated. Then, we stratified by region and constructed three multivariate regression models using Cox proportional hazards regression to examine whether there were significant differences in risk factors for depression and their risks between regions. Thirdly, we evaluated the relationship between each chronic disease and depression by enrolling 13 chronic diseases in a Cox proportional hazards regression model after adjusting for the effects of all covariates. Finally, we explored whether there were interaction effects of social activity participation, age, and chronic disease status on the incidence of depression by dividing the sample population into four categories, namely, “Middle-aged–Have social activity,” “Middle-aged–No social activity,” “Older adult–Have social activity,” and “Older adult–No social activity”. In the Cox proportional hazards regression, when the *HR* is greater than 1 and the 95% *CI* does not include 1, it indicates a higher risk of depression in this group than in the control group, while the opposite is true for *HR* less than 1. The SPSS 26.0 (SPSS Inc., Chicago, IL, United States) was used for all statistical analyzes in this study, and differences were considered statistically significant at *p* < 0.05.

## 3. Result

### 3.1. Sample characteristics

There was a total of 7,163 participants aged 45 years or older included in this study, of whom 3,793 (53.0%) were male and 3,370 (47.0%) were female. The age range was 45–93 years, with a mean age of 58.4 ± 9.0 years (median age 57 years), and the middle-aged population under 60 years was 58.6%. Most participants were characterized as having an elementary school education or less (62.3%), being married (90.8%), having health insurance (94.2%), and having no income (80.6%). At a mean follow-up of 5.08 ± 2.08 years, depression was reported in 2951 (41.2%; 95% *CI*: 40.1–42.3%) of 7,163 participants, with a cumulative incidence of 35.0% (95% *CI*: 33.5–36.5%) in male and 48.2% (95% *CI*: 46.5–49.9%) in female. The baseline chronic disease prevalence in the sample population was 60.7%, with 29.4% of patients with multiple diseases and 12.1% with three or more chronic diseases. The prevalence of chronic diseases among middle-aged people under 60 years old was 56.1, and 67.3% for those aged 60 years and above. The highest prevalence of chronic diseases at baseline were arthritis or rheumatism, hypertension, and stomach or other digestive diseases, with prevalence rates of 26.9, 21.8, and 18.0%, respectively.

### 3.2. Relationship between covariates and depression

The association of each covariate with the hazard of developing depression was estimated using the univariate Cox proportional hazards regression model. The results suggested that all variables except age and health insurance were significantly related to depression (*p* < 0.05). The middle-aged and older adult Chinese characterized as female, lower education, widowed, not getting enough sleep, never smoking or drinking, lacking social activities, no income, low BMI, and less developed regions (Central and Western) were more likely to develop depression (Table 1).

**Table 1:** Baseline characteristics and incidence of depression in 7163 participants, *n* (%).

Variables	Total	Depression situation		Crude *HR* (95% *CI*)	*p*
		No	Yes		
Total	7,163 (100)	4,212 (58.8)	2,951 (41.2)		
Age (year)					
<60	4,201 (58.6)	2,451 (58.3)	1750 (41.7)	1	
≥60	2,962 (41.4)	1761 (59.5)	1,201 (40.5)	1.027 (0.954,1.105)	0.483
Gender					
Male	3,793 (53.0)	2,466 (65.0)	1,327 (35.0)	1	
Female	3,370 (47.0)	1746 (51.8)	1,624 (48.2)	1.451 (1.349,1.560)	<0.001
Highest level of education					
High school and above	1,002 (14.0)	724 (72.3)	278 (27.7)	1	
Middle school	1,698 (23.7)	1,080 (63.6)	618 (36.4)	1.340 (1.164,1.544)	<0.001
Primary school and below	4,463 (62.3)	2,408 (54.0)	2055 (46.0)	1.802 (1.590,2.043)	<0.001
Marital status					
Married	6,506 (90.8)	3,845 (59.1)	2,661 (40.9)	1	
Divorced	69 (1.0)	44 (63.8)	25 (36.2)	0.870 (0.586,1.289)	0.487
Widowed	551 (7.7)	305 (55.4)	246 (44.6)	1.222 (1.072,1.392)	0.003
Unmarried	37 (0.5)	18 (48.6)	19 (51.4)	1.321 (0.841,2.074)	0.227
Health insurance status					
Yes	6,744 (94.2)	3,982 (59.0)	2,762 (41.0)	1	
No	419 (5.8)	230 (54.9)	189 (45.1)	1.157 (0.999,1.341)	0.052
Nighttime sleep duration (hour)					
>8	2,467 (34.4)	1,513 (61.3)	954 (38.7)	1	
5–8	4,033 (56.3)	2,394 (59.4)	1,639 (40.6)	1.006 (0.984,1.154)	0.119
<5	663 (9.3)	305 (46.0)	358 (54.0)	1.569 (1.389,1.772)	<0.001
Drinking status					
>Once/month	2012 (28.1)	1,307 (65.0)	705 (35.0)	1	
<Once/month	602 (8.4)	375 (62.3)	227 (37.7)	1.074 (0.925,1.247)	0.349
Never	4,549 (63.5)	2,530 (55.6)	2019 (44.4)	1.326 (1.217,1.445)	<0.001
Smoking status					
Current smoker	2,388 (33.3)	1,500 (62.8)	888 (37.2)	1	
Former smoker	670 (9.4)	436 (65.1)	234 (34.9)	0.950 (0.823,1.098)	0.490
Never smoked	4,105 (57.3)	2,276 (55.4)	1829 (44.6)	1.233 (1.138,1.335)	<0.001
Social activity participation					
Yes	3,889 (54.3)	2,418 (62.2)	1,471 (37.8)	1	
No	3,274 (45.7)	1794 (54.8)	1,480 (45.2)	1.236 (1.150,1.328)	<0.001
Income status					
Yes	1,393 (19.4)	959 (68.8)	434 (31.2)	1	
No	5,770 (80.6)	3,253 (56.4)	2,517 (43.6)	1.497 (1.352,1.658)	<0.001
BMI (kg/m^2^)					
18.5 ~ 24	3,712 (51.8)	2,195 (59.1)	1,517 (40.9)	1	
<18.5	359 (5.0)	191 (53.2)	168 (46.8)	1.265 (1.079,1.484)	0.044
24 ~ 28	2,208 (30.8)	1,301 (58.9)	907 (41.1)	0.984 (0.906,1.069)	0.703
>28	884 (12.3)	525 (59.4)	359 (40.6)	0.981 (0.874,1.100)	0.738
Region					
Eastern	2,472 (34.5)	1,631 (66.0)	841 (34.0)	1	
Central	2,629 (36.7)	1,527 (58.1)	1,102 (41.9)	1.252 (1.144,1.369)	<0.001
Western	2062 (28.8)	1,054 (51.1)	1,008 (48.9)	1.536 (1.402,1.684)	<0.001

### 3.3. Relationship between chronic diseases and depression

**Figure 2:**
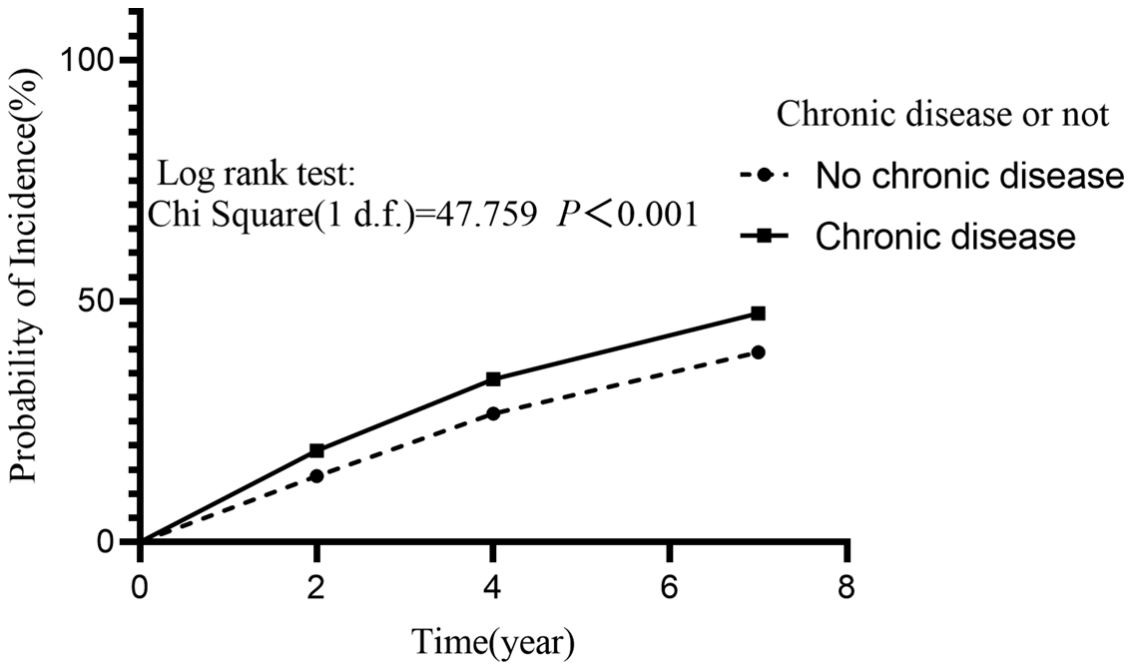
Effect of participant’s baseline chronic disease status on the incidence of depression. The incidence of depression was significantly higher in participants with chronic disease than in those without chronic disease at baseline (*HR* = 1.330, 95% *CI*: 1.227–1.442, *p* < 0.001).

**Figure 3:**
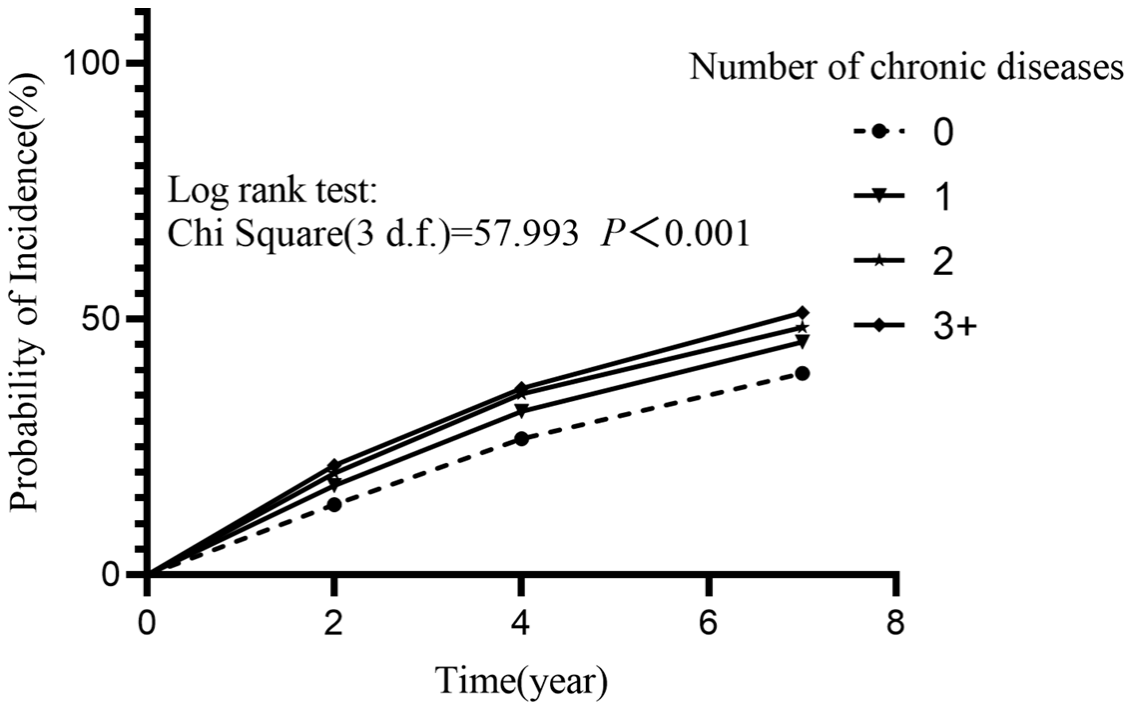
Effect of the number of participants’ baseline chronic diseases on the incidence of depression. The incidence of depression increased significantly with the increasing number of chronic diseases at baseline (*p*-trend <0.001). Relative to participants without chronic diseases, the risk of depression for participants with one chronic disease: *HR* = 1.203, 95% *CI*: 1.101–1.313; for participants with two chronic diseases: *HR* = 1.316, 95% *CI*: 1.187–1.459; and for participants with three or more chronic diseases: *HR* = 1.406, 95% *CI*: 1.253–1.577. All *p* < 0.001.

**Figure 4:**
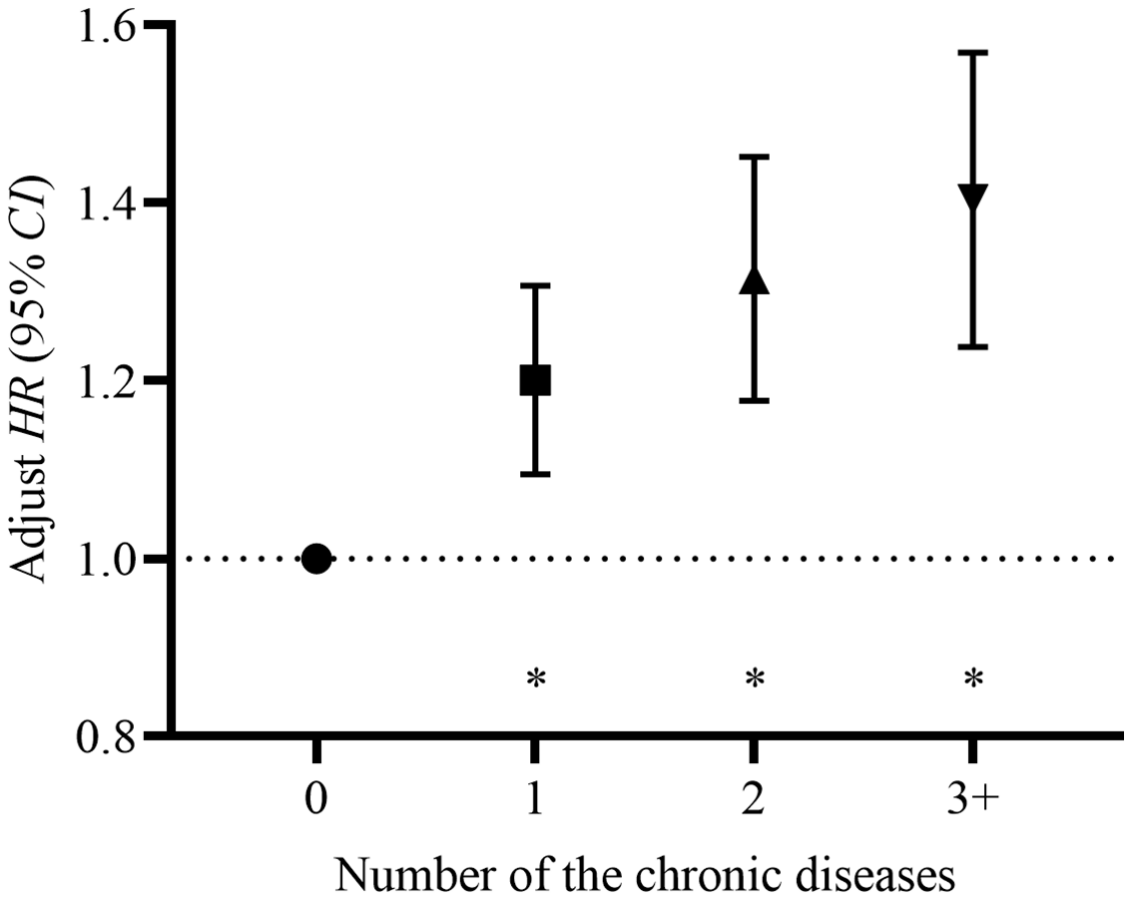
Adjust hazard (*HR*) and its 95% confidence interval (95% *CI*) of the number of the chronic diseases at baseline. The Cox proportional hazards regression model was adjusted for age, gender, highest level of education, marital status, health insurance, nighttime sleep duration, drinking status, smoking status, social activity participation, income status, and BMI, *p < 0.001. The risk of developing depression increased significantly with the number of chronic diseases at baseline (*p*-trend < 0.001). Relative to participants without chronic diseases, the risk of depression for participants with one chronic disease: *HR*_adjust_ = 1.197, 95% *CI*: 1.096–1.308; for participants with two chronic diseases: *HR*_adjust_ = 1.310, 95% *CI*: 1.180–1.454; and for participants with three or more chronic diseases: *HR*_adjust_ = 1.397, 95% *CI*: 1.241–1.572. All *p* < 0.001.

We evaluated the association between baseline chronic disease and depression by employing the Kaplan–Meier method and Cox proportional hazards regression models. Findings revealed that chronic disease at baseline was at a significantly higher risk of developing depression relative to those who did not have any chronic disease (Log-rank test: *χ^2^* = 47.759, *p* < 0.001) (Figure 2). Furthermore, the risk of developing depression in the population increased significantly with the number of chronic diseases (Log-rank test: *χ^2^* = 57.993, *p* < 0.001, *p*-trend < 0.001) (Figure 3). After adjusting for the impact of covariates in the multivariate Cox proportional hazards regression model, we found that as the number of chronic diseases increased, the risk of developing depression increased (*p-*trend < 0.001) (Figure 4).

After stratifying by region, the current study found some differences in the risk relationship between chronic diseases and depression among the Eastern, Central, and Western populations, as well as some differences in risk factors for depression among the regions. In terms of differences in risk factors, the risk of depression among middle-aged and older adult people in the Eastern region was influenced by health insurance status and BMI, while significant differences were observed between gender, social activity participation, and income status in the Central and Western regions (Table 2).

**Table 2:** Association between each variable and depression after stratification by region.

Variables	Model 1 (Eastern)		Model 2 (Central)		Model 3 (Western)	
	*HR* (95% *CI*)	*p*	*HR* (95% *CI*)	*p*	*HR* (95% *CI*)	*p*
Age (year)						
<60	1		1		1	
≥60	0.864 (0.741, 1.007)	0.062	0.865 (0.757, 0.989)	0.033	0.927 (0.808, 1.064)	0.284
Gender						
Male	1		1		1	
Female	1.134 (0.923, 1.392)	0.231	1.421 (1.191, 1.696)	<0.001	1.447 (1.200, 1.744)	<0.001
Highest level of education						
High school and above	1		1		1	
Middle school	1.586 (1.204, 2.089)	0.001	1.137 (0.917, 1.410)	0.241	1.195 (0.911, 1.567)	0.198
Primary school and below	1.821 (1.413, 2.347)	<0.001	1.410 (1.155, 1.721)	<0.001	1.576 (1.229, 2.021)	<0.001
Marital status						
Married	1		1		1	
Divorced	1.019 (0.525, 1.980)	0.956	0.888 (0.359, 1.997)	0.775	1.070 (0.571, 2.006)	0.833
Widowed	1.234 (0.960, 1.610)	0.099	1.064 (0.847, 1.336)	0.596	0.966 (0.768, 1.215)	0.765
Unmarried	1.411 (0.695, 2.864)	0.341	1.107 (0.411, 2.980)	0.841	1.652 (0.775, 3.522)	0.194
Health insurance status						
Yes	1		1		1	
No	1.363 (1.032, 1.802)	0.029	0.935 (0.728, 1.201)	0.935	1.145 (0.893, 1.468)	0.285
Nighttime sleep duration (hour)						
>8	1		1		1	
5–8	1.090 (0.939, 1.265)	0.259	1.084 (0.950, 1.236)	0.229	1.125 (0.980, 1.293)	0.095
<5	1.599 (1.242, 2.058)	<0.001	1.377 (1.129, 1.680)	0.002	1.305 (1.067, 1.596)	0.010
Drinking status						
>Once/month	1		1		1	
<Once/month	1.042 (0.782, 1.390)	0.777	1.071 (0.836, 1.372)	0.588	1.023 (0.791, 1.324)	0.860
Never	1.186 (0.981, 1.435)	0.078	1.120 (0.948, 1.323)	0.181	0.950 (0.804, 1.122)	0.545
Smoking status						
Current smoker	1		1		1	
Former smoker	0.916 (0.707, 1.187)	0.508	0.883 (0.694, 1.125)	0.315	1.107 (0.854, 1.434)	0.443
Never smoked	1.001 (0.816, 1.230)	0.959	0.916 (0.768, 1.092)	0.327	0.962 (0.798, 1.161)	0.688
Social activity participation						
Yes	1		1		1	
No	1.084 (0.945, 1.245)	0.249	1.136 (1.007, 1.281)	0.038	1.218 (1.072, 1.383)	0.003
Income status						
Yes	1		1		1	
No	1.117 (0.941, 1.327)	0.207	1.203 (1.000, 1.447)	0.050	1.265 (1.002, 1.597)	0.048
BMI (kg/m^2^)						
18.5 ~ 24	1		1		1	
<18.5	1.235 (0.898, 1.697)	0.194	1.172 (0.888, 1.545)	0.262	1.142 (0.887, 1.472)	0.304
24 ~ 28	1.015 (0.900, 1.226)	0.530	0.977 (0.852, 1.121)	0.740	0.962 (0.826, 1.121)	0.623
>28	0.770 (0.615, 0.964)	0.023	0.973 (0.805, 1.175)	0.775	1.006 (0.814, 1.242)	0.958
Number of chronic diseases						
0	1		1		1	
1	1.156 (0.982, 1.359)	0.081	1.208 (1.042, 1.400)	0.012	1.230 (1.056, 1.433)	0.008
2	1.356 (1.108, 1.660)	0.003	1.416 (1.193, 1.681)	<0.001	1.204 (1.010, 1.435)	0.038
3+	1.367 (1.081, 1.728)	0.009	1.434 (1.188, 1.730)	<0.001	1.385 (1.129, 1.699)	0.002

**Figure 5:**
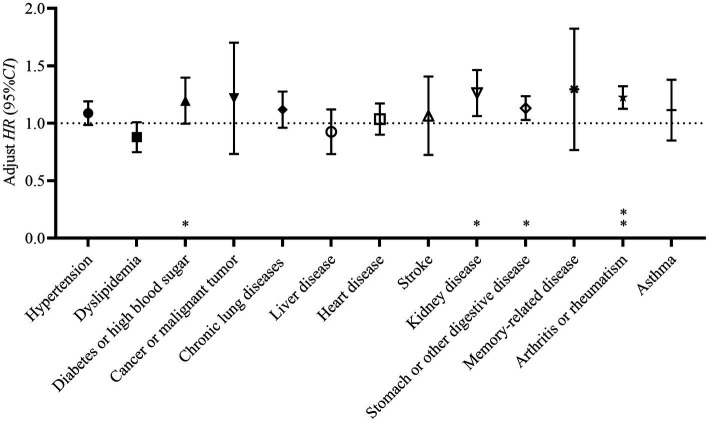
Adjust hazard (*HR*) and its 95% confidence interval (95% *CI*) of having different kinds of chronic diseases at baseline. The Cox proportional hazards regression model was adjusted for age, gender, highest level of education, marital status, health insurance, nighttime sleep duration, drinking status, smoking status, social activity participation, income status, BMI and other types of chronic diseases, *p < 0.05; **p < 0.001. Having the diabetes or high blood sugar, kidney disease, stomach or other digestive diseases, and arthritis or rheumatism at baseline significantly increased the risk of developing depression in participants. Diabetes or high blood sugar: *HR*_adjust_ = 1.185, 95% *CI*: 1.001–1.403; Kidney disease: *HR*_adjust_ = 1.252, 95% *CI*: 1.067–1.468; Stomach or other digestive disease: *HR*_adjust_ = 1.128, 95% *CI*: 1.029–1.236; Arthritis or rheumatism: *HR*_adjust_ = 1.221, 95% *CI*: 1.127–1.323.

Furthermore, our study included 13 kinds of chronic diseases in the covariate-adjusted multivariate Cox proportional hazards regression model, to examine each of the chronic diseases on depression. The results of the analysis showed that diabetes or high blood sugar, kidney disease, stomach or other digestive diseases, and arthritis or rheumatism significantly raised the risk of depression in our study population (Figure 5).

### 3.4. Interaction effects of social activity participation, age, and chronic disease status on the incidence of depression

**Figure 6:**
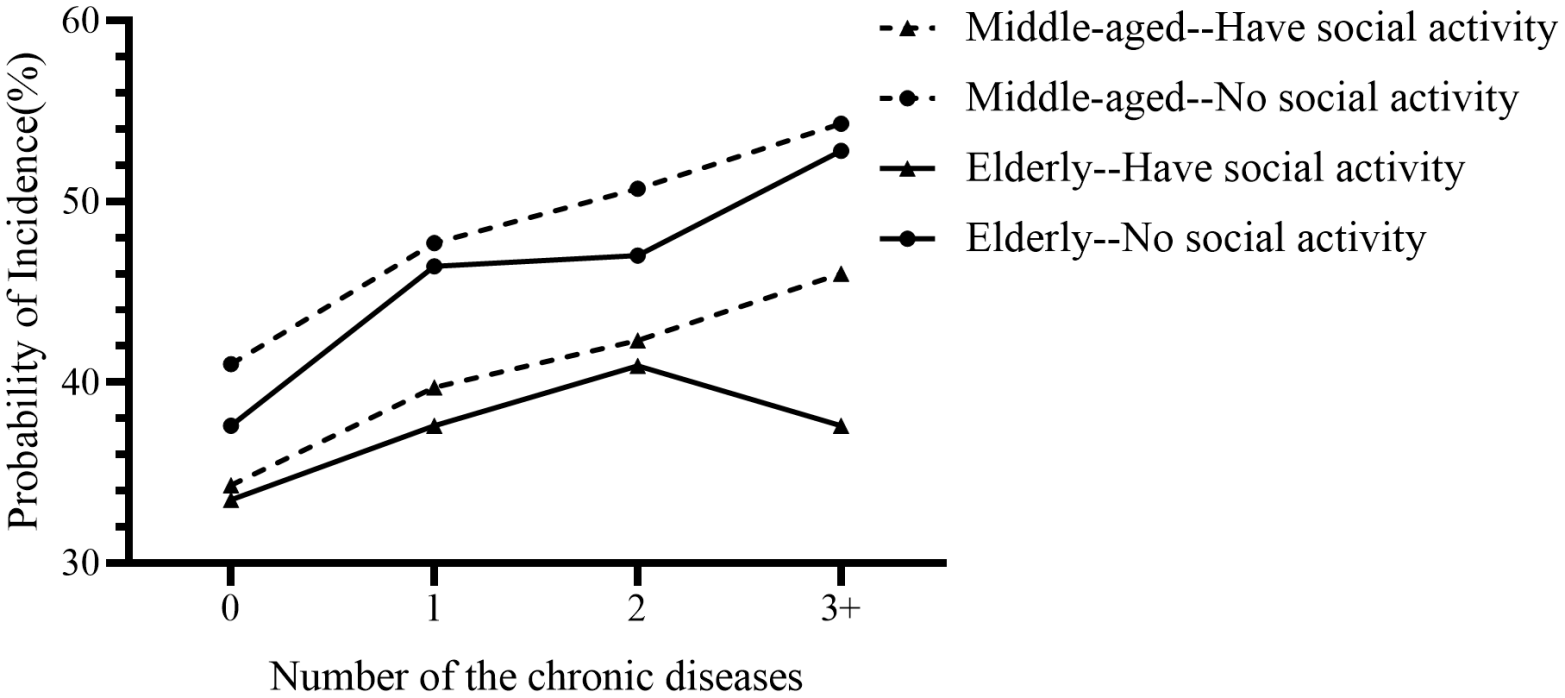
Interaction effects of social activity participation, age, and chronic disease status on the prevalence of depression. The prevalence of depression was higher in both middle-aged and older adult participants who had no social activity than in those who had social activity, and both in the had social activity and had no social activity groups, the prevalence of depression was lower in the older adult than in middle-aged adults.

The results of the interaction effects analysis indicated that there were interaction effects of chronic disease status, age, and social activity participation on the incidence of depression. Specifically, the incidence of depression was lower among individuals who engaged in social activities, both in the middle-aged group (<60 years) and older adult group (≥60 years); and the incidence of depression was significantly higher in the middle-aged group compared to the older adult group, regardless of their participation in social activities (Figure 6).

## 4. Discussion

At present, the CHARLS is one of the most representative sociodemographic investigations in China, with its long-term period, broad survey scope, and various research variables. In the current study, we analyzed the relationship between the chronic disease and depression among middle-aged and older adult people aged 45 years and older in China using the cohort data from CHARLS 2011–2018. We observed that participants with chronic diseases were at higher risk of depression, and the risk was directly proportional to the number of chronic diseases. Further analysis of chronic conditions revealed that suffering from diabetes or high blood sugar, kidney disease, stomach or other digestive diseases, and arthritis/rheumatism increased the risk of depression significantly. In addition, there are some differences in the influencing factors of depression and its risk between different regions. In summary, our study illustrates the crucial role of chronic diseases in the occurrence of depression, and it guides the prevention and treatment of depression in middle-aged and older adult adults in China.

### 4.1. The prevalence of depression in Chinese middle-aged and older adult populations is dismal

After seven years of follow-up, 41.2% of the sample population reported depression, which is higher than several previous studies on depression (9, 14, 43), suggesting that the status of depression in the Chinese population is not optimistic. However, the studies showed that the outpatient rate of depression is extremely poor, with only 5% of middle-aged and older adult people with depression being aware of their condition and 2% consistently seeking treatment (43). The middle-aged and older adult Chinese populations are strongly influenced by traditional concepts and generally have a low level of education, so they did not form a correct awareness of depression, and consider it a “shame” to suffer from depression. Researches have shown that older and lower-educated people hold higher levels of stigma about depression (44, 45). The stigma attached to depression can seriously hinder the recovery of the social functioning of patients, making them more vulnerable to misunderstanding and discrimination (46), thus reducing their willingness to seek medical care and increasing their risk of suicide (47, 48).

### 4.2. There was a dose–response relationship between the number of chronic diseases and the risk of depression

According to our study, suffering from different levels of chronic disease increased the risk of developing depression among participants. A similar study reported that chronic diseases were related to a significantly higher risk of depression (*HR* = 1.38), and this risk increased with the number of chronic diseases (9). Additionally, a meta-analysis found that the people with multiple diseases had twice the risk of depression as those without multiple diseases (*RR* = 2.13, *p* < 0.001), and the odds of depression were 45% higher for each additional chronic disease compared with those without chronic disease (*OR* = 1.45, *p* < 0.001) (25). We supposed that chronic diseases are difficult to cure for a long time, and will cause a substantial psychological and physiological burden to patients, and some of them even resist the long-term tedious disease treatment. When suffering from multiple chronic diseases, the negative emotions may get worse with the number of chronic diseases they suffer from, and the accumulation of negative emotions over time will most likely lead to depression. Besides, one of the contributors to the increased vulnerability to depression in patients with chronic diseases is economic factors. Most chronic diseases are long-lasting, and some are even incurable, requiring lifelong treatment, which will impose a tremendous financial burden and additional labor losses on patients and their families (49, 50). It has been found that 63.96% of the total expected medical expenses for the older adult in rural areas of China are for chronic diseases (50), and the enormous medical expenses will increase the psychological stress of the patients and lead to depression.

### 4.3. Certain specific diseases (diabetes, kidney disease, stomach or other digestive disease, and arthritis or rheumatism) increase the risk of depression significantly

Furthermore, our study found that patients suffering from diabetes or high blood sugar, kidney disease, stomach or other digestive diseases, and arthritis or rheumatism has a significantly increased risk of depression, and the highest risk was found for kidney disease. Zheng also discovered that chronic kidney disease raised the risk of depression in the middle-aged and older adult population (*OR* = 1.48, *p* < 0.05) (21). We supposed that chronic kidney disease has a recurrent course and low curative rate, and treatment such as hemodialysis and peritoneal dialysis is required. Patients must endure the devastating effects of the complex disease treatment process and side effects on their family, work employment, mental health, and freedom for a long time (51–53), and long-term poor emotions will develop depression. It is also important to note that patients with arthritis or rheumatism face a high risk of depression because arthritis or rheumatism causes persistent pain, resulting in loss of appetite, mobility, poor sleep, and even loss of function, which can significantly affect patients’ daily life and lead to the anxiety and depression (54), about 30% of patients with rheumatoid arthritis developed depression within 5 years of disease diagnosis (55). Additionally, studies have shown particular biological interactions between the immune system and the central nervous system (56). Some inflammatory factors [such as interleukin-1 (IL-1), tumor necrosis factor- (TNF) and IL6] enter the central nervous system through humoral or neuronal pathways and disrupt its normal functioning, resulting in the development of mental disorders (57, 58), and these inflammatory factors have also been demonstrated to be associated with the duration and severity of depression (57). Furthermore, through being implicated in the etiology of pain and weariness, these inflammatory factors indirectly contribute to depression (57).

### 4.4. The risk of depression is significantly higher among female than male in the middle-aged and older adult population

Through univariate Cox proportional hazards regression model analysis, the current study found that the risk of depression was significantly higher among females than males, which was consistent with the findings of several previous studies (9, 59–62). The main reasons included the higher average life expectancy of women compared to men, thus resulting in a higher rate of widowhood, and the difficulty for female seniors to adapt to life and emotional changes in the short term, which leads to feelings of loneliness (29). Furthermore, the women faced fewer education opportunities, poor economic situation, and poor social competitiveness compared to the men, which may also contribute to a greater risk of depression in older adult women. Moreover, due to the rapid changes in their physical condition and hormone levels, middle-aged women are prone to the “menopause syndrome” during and after perimenopause, resulting in symptoms such as menstrual disorders, dizziness, body pain and hot flashes. Also, the menopausal women are more likely to develop sleep disorders and sexual dysfunction, which can seriously reduce the quality of life and increase the risk of depression (63, 64). For this reason, we should be more concerned about the mental health of women and provide them with more support in disease prevention and social security.

### 4.5. Several sociodemographic and lifestyle factors have significant effects on the risk of depression

Moreover, several sociodemographic and lifestyle factors, such as sleep duration, participation in social activities, and socioeconomic standing, have a substantial influence on depression. Our research revealed that those who get insufficient sleep had a higher risk of depression (<5 h/d: Crude HR = 1.569). That is because adequate sleep is a necessary prerequisite for maintaining physiological health, and when lacking sufficient hours of sleep, it is difficult to maintain the homeostasis of the organism and prone to various diseases (65, 66). Compared to youthful people, middle-aged and older adult adults generally have a shorter sleep duration and poorer sleep quality (67–69), thus being more vulnerable to melancholy. Previous research has demonstrated that reduced sleep duration over short periods of time is associated with impaired emotional functioning, memory, and attention (70), and that changes in sleep duration may lead to alterations or disruptions of circadian rhythms, as well as circadian rhythms may regulate mood, according to some evidence (71, 72). Additionally, poor social activity is a significant risk factor for depression, and numerous studies have demonstrated a strong negative correlation between depression and social activity (73–75), the results of the interaction effects analysis in the current study also showed that whether in the middle-aged or older adult population, the incidence of depression was higher in the had no social activity group than the had social activity group. On the one hand, abundant social activities may increase the psychological satisfaction of life, as well as their sense of well-being and social support (76, 77), thereby alleviating the depressive state. On the other hand, social activities enable the older adult to engage in more physical activity, which can significantly enhance their physical condition and cognitive ability (77, 78) and decrease the risk of depression (79, 80). In addition, economic status exerts a greater influence on depression, with the current study revealing a substantially higher risk of depression in those without income than in those with income (Crude HR = 1.497). Inferior socioeconomic status impairs the capacity to process negative emotions, resulting in increased cognitive and negative emotional vulnerability (16, 81), and individuals with poorer economic status often struggle to access long-term, professional mental health services, leaving them without depression diagnosis and treatment (81). Apart from personal economic status, community economic status is equally crucial for health (82–85), and the double jeopardy hypothesis suggests that people living in communities with lower socioeconomic status are less healthy than those living in regions with higher socioeconomic status because they are more likely to be exposed to a greater lack of public health services and community health resources (86–88). Therefore, in terms of health policy, we recommend that government departments prioritize the mental health of those residing in lower socioeconomic status regions, who are entitled to receive community-based health care services regardless of their individual socioeconomic status.

### 4.6. China should take effective measures to protect the mental health of middle-aged and older adult people, especially those suffering from chronic diseases

In summary, the middle-aged and older adult with chronic diseases were more likely to develop depression, and these findings provide targets for future aging research and public health interventions in China, where current policies need to increase investment and attention to mental health support for patients with chronic diseases. It is essential to provide extensive health education to those who are generally less educated, to make them correctly understand depression, and to strengthen mental health education and knowledge dissemination among social workers, volunteers and family members to cultivate a positive mindset and an optimistic worldview among the middle-aged and older adult. The public also needs to be guided to reduce disease discrimination, help patients with depression build confidence, and increase the rate of depression diagnosis. In addition, the government should take a series of measures to safeguard the mental health of patients with chronic diseases, such as increasing social benefits (e.g., pensions), improving their living standards, and scheduling frequent follow-up visits with patients. In terms of preventing chronic diseases, efforts should be increased to prevent and treat chronic diseases, encourage regular medical checkups for middle and older adults, and detect and treat various chronic diseases at an early stage. Furthermore, it is necessary to strengthen the collaboration between medical institutions and family solidarity to improve the quality of treatment for middle-aged and older adult patients and reduce the possibility of transformation of chronic diseases into depression; as well as to expand the medical insurance coverage of chronic disease drugs to reduce the economic burden of chronic disease patients, thus indirectly improving the mental health status of the sick population. Another objective is required to focus on screening for depression in people with chronic diseases, such as free depression screening in primary hospitals, as well as an early and inexpensive intervention treatment for people with the disease to prevent its deterioration and reduce the suicide rate in people with depression. All in all, China should pay more attention to the mental health problems of middle-aged and older adult people, improve primary medical conditions and social support services, and provide a full range of treatment, management and rehabilitation services for patients through the construction of health information technology in multiple sites. At the same time, targeted preventive and therapeutic measures are taken for different types of chronic diseases to reduce the negative effects of chronic diseases and decrease the incidence of depression. In addition, our study found that there were some differences in risk factors for depression in different regions, and relevant authorities should consider these factors when formulating depression prevention and treatment policies and adopting appropriate countermeasures for populations in different regions.

There are also some limitations to our study. First, we did not consider possible changes in variables with follow-up time when examining the relationship between each baseline characteristic and depression. Secondly, the chronic diseases and depression status investigations were based on participants’ self-reports, which may generate various degrees of recall bias and thus deviance in assessing the relationship between chronic diseases and depression. In addition, the covariates included in the present study were based on extensive literature reading and expert consultation, certain variables with implications for depression may not have been considered in this study. Finally, the sample population of the current study excluded those who resided in orphanages and older adult institutions, whose mental health status may have been worse than that of the older adult who lived at home, during the original data sampling phase.

## 5. Conclusion

The current study revealed a dose–response relationship between chronic diseases and the risk of depression in the middle-aged and older adult Chinese population, as well as the significant impact of certain diseases on the risk of depression. It could serve as a guide for the prevention and treatment of depression in this population, and relevant authorities should prioritize the mental health of people with chronic diseases when formulating disease prevention and treatment policies to reduce the incidence and disease burden of depression and improve the quality of life of the middle-aged and older adult population. Compared to other studies of the same type, our study features a large sample size, a lengthy follow-up period, and the use of the most recent CHARLS data, allowing for a more accurate assessment of the association between chronic disorders and depression. However, the focus of the current study was on the number of chronic diseases and the effect of specific chronic diseases on depression, without exploring the effect of the combination of different types of chronic diseases and their interaction on depression, a topic that will require further investigation in the future.

## Data availability statement

The datasets presented in this study can be found in online repositories. The names of the repository/repositories and accession number(s) can be found at: http://charls.pku.edu.cn. The database is free and open to scholars worldwide.

## Ethics statement

The studies involving human participants were reviewed and approved by CHARLS was ethically approved by the Ethics Review Board of the Peking University (approval number: IRB00001052-11015), and each respondent signed an informed consent form. Written informed consent for participation was not required for this study in accordance with the national legislation and the institutional requirements. Written informed consent was obtained from the individual(s) for the publication of any potentially identifiable images or data included in this article.

## Author contributions

PZ, YL, and XY designed the research program. JP and WG performed the statistical analysis. QL processed the figures and table. SW and YY wrote the manuscript. All authors contributed to the article and approved the submitted version.

## Funding

This work was supported by the special scientific research project for health care of the People’s Liberation Army of China (21BJZ39), and the military medical research project, the General Hospital of Western Theater Command, the People’s Liberation Army of China (2021-XZYG-A14). These sponsors had no role in the design implementation, data analysis, and report writing of this study.

## Conflict of interest

The authors declare that the research was conducted in the absence of any commercial or financial relationships that could be construed as a potential conflict of interest.

## Publisher’s note

All claims expressed in this article are solely those of the authors and do not necessarily represent those of their affiliated organizations, or those of the publisher, the editors and the reviewers. Any product that may be evaluated in this article, or claim that may be made by its manufacturer, is not guaranteed or endorsed by the publisher.
