# Supplementary material for: Association between chronic diseases and depression in the middle-aged and older adult Chinese population—a seven-year follow-up study based on CHARLS
Source: Front Public Health. 2023 Jul 20;11:1176669. doi: 10.3389/fpubh.2023.1176669 (PMC10403076; doi:10.3389/fpubh.2023.1176669)
Supplement: Supplementary file 1 [file Data_Sheet_1.docx]

**Appendix 1 The code for all variables and their problem descriptions**

| **Variable** | **Code** | **Question description** | **Answer** |
| --- | --- | --- | --- |
| Gender | BA000_W2_3 | Interviewer record the Respondent’s gender | 1. Male  2. Female |
| Age | BA002 | What’s your actual date of birth? |  |
| Highest level of education | BD001_W2_4 | What’s the highest level of education your have now (not including adult education)? | 1. No formal education (illiterate)  2. Did not finish primary school  3. Sishu/home school  4. Elementary school  5. Middle school  6. High school  7. Vocational school  8. Two-/Three-Year College/Associate degree  9. Four-Year College/Bachelor’s degree  10. Master’s degree  11. Doctoral degree/Ph.D. |
| Marital status | BE001 | What is your marital status? | 1. Married and live with spouse  2. Married but don’t living with spouse temporarily for reasons such as work  3. Separated, don’t live together as a couple anymore  4. Divorced  5. Widowed  6. Never married |
| Health insurance status | EA001_W4 | Are you the policy holder/primary beneficiary of any of the types of health insurance listed below? (circle all that apply) | 1. Urban employee medical insurance (yi-bao)  2. Urban and rural resident medical insurance (integrated urban resident medical insurance and new rural cooperative medical insurance)  3. Urban resident medical insurance  4. New rural cooperative medical insurance (he-zuo-yi-liao)  5. Government medical insurance  6. Medical aid  7. Private medical insurance: purchased by work unit  8. Private medical insurance: purchased by individual  9. Urban non-employed persons’s health insurance  10. Long-term care insurance  11. Other medical insurance  12. No insurance |
| Nighttime sleep duration | DA049 | During the past month, how many hours of actual sleep did you get at night (average hours for one night)? (This may be shorter than the number of hours you spend in bed.) |  |
| Smoking status | DA059 | Have you ever chewed tobacco, smoked a pipe, smoked self-rolled cigarettes, or smoked cigarettes/cigars? | 1. Yes  2. No |
|  | DA061 | Do you still have the habit or have you totally quit? | 1. Still have  2. Quit |
| Drinking status | DA067 | Did you drink any alcoholic beverages, such as beer, wine, or liquor in the past year? How often? | 1. Drink more than once a month  2. Drink but less than once a month  3. None of these |
| Social activity participation | DA056 | Have you done any of these activities in the last month? (Check all that apply) | 1. Interacted with friends  2. Played Ma-jong, played chess, played cards, or went to community club  3. Provided help to family, friends, or neighbors who do not live with you  4. Went to a sport, social, or other kind of club  5. Took part in a community-related organization  6. Done voluntary or charity work  7. Cared for a sick or disabled adult who does not live with you  8. Attended an educational or training course  9. Stock investment  10. Used the Internet  11. Other  12. None of these |
| Income status | GA001 | Did you receive any wage and bonus income in the past year? | 1. Yes  2. No |
| Height | PI001 | Can you understand the measurement method and are you willing to participate in this measurement? | 1. YES  2. No |
| Weight | PL002 | Can you understand the measurement method and are you willing to participate in this measurement? | 1. Yes  2. No |
| Chronic disease | DA007 | Have you been diagnosed with [conditions listed below, read one by one] by a doctor? | 1. Hypertension  2. Dyslipidemia  3. Diabetes or high blood sugar  4. Cancer or malignant tumor  5. Chronic lung diseases  6. Liver disease  7. Heart attack  8. Stroke  9. Kidney disease  10. Stomach or other digestive diseases  11. Memory-related disease  12. Arthritis or rheumatism  13. Asthma |
| Depression | DC009 | I was bothered by things that don’t usually bother me | 1. Rarely or none of the time  2. Some or a little of the time  3. Occasionally or a moderate amount of the time  4. Most or all of the time  8. Do not know  9. Refuse to answer |
|  | DC010 | I had trouble keeping my mind on what I was doing |  |
|  | DC011 | I felt depressed |  |
|  | DC012 | I felt everything I did was an effort |  |
|  | DC013 | I felt hopeful about the future |  |
|  | DC014 | I felt fearful |  |
|  | DC015 | My sleep was restless |  |
|  | DC016 | I was happy |  |
|  | DC017 | I felt lonely |  |
|  | DC018 | I could not get “going” |  |
